# A stable solid-like water at normal condition

**DOI:** 10.1038/s41598-026-42682-x

**Published:** 2026-05-07

**Authors:** An Wei-qing, Yue Xiang-an, Zou Ji-rui

**Affiliations:** 1 State Key Laboratory of Petroleum Resources and Prospecting, University of Petroleum (Beijing), Beijing, 102249 China; 2 College of Petroleum Engineering, China University of Petroleum-Beijing, Beijing, 102249 China; 3 College of Energy Innovation, China University of Petroleum-Beijing, Beijing, 102249 China; 4 Research lnstitute Exploration & Development of Xinjiang Oilfield Company, CNPC, Karamay, Xinjiang China

**Keywords:** Solid-like water, SiO_2_ microtubule, Normal condition, molecular kinetic energy, Chemistry, Materials science, Physics

## Abstract

The physical state of water under extreme confinement can deviate from bulk behavior, yet such effects are typically considered limited to the sub-10 nm regime. Here we report a stable solid-like water phase under ambient conditions in SiO₂ microtubules spanning the submicron–micron scale. The confined water shows pronounced solid-like behavior: it can be sectioned by focused ion beam (FIB) milling and deforms plastically under stress rather than flowing, while maintaining a stable morphology from − 20 to 90 °C and from high vacuum (10⁻⁵ Pa) to atmospheric pressure. ¹H NMR, Raman, and IR spectra indicate strongly restricted molecular dynamics and a modified hydrogen-bonding environment distinct from bulk liquid water and crystalline ice. ¹H NMR quantification and surface-chemistry controls further identify an exceptionally high density of inner-wall silanol (SiOH) groups as the key factor governing formation and stability, with clear pH dependence and reversible transitions. These findings highlight a mesoscale confinement regime dominated by interfacial chemistry.

**Supplementary Information:**

The online version contains supplementary material available at 10.1038/s41598-026-42682-x.

## Introduction

Water is the most ubiquitous substance on Earth, and its liquid phase has been extensively studied. Nevertheless, a variety of anomalous water states have been reported under special physicochemical conditions, including highly viscous water layers on mineral surfaces^1^ and square ice stabilized in nanoconfined environments under ambient conditions^2^.

Over the past two decades, increasing attention has been devoted to the structure and properties of water at silica interfaces and within nanoconfined spaces. Most previous studies have relied on molecular dynamics simulations to investigate these systems^3–13^. A central outcome of these investigations is the identification of two distinct interfacial water states: a “liquid-like” state and an “ice-like” state, reflecting different hydrogen-bonding configurations at solid–liquid interfaces.

In addition to simulation-based studies, several experimental investigations have reported anomalous water behavior at silica interfaces and in nanoconfined environments. Klein et al. measured viscous forces of water confined between mica surfaces using a surface force apparatus and observed solid-like viscosity when the film thickness was reduced to a few molecular layers^1^. Crupi et al. employed incoherent quasi-elastic neutron scattering and Fourier-transform infrared spectroscopy to probe water confined in nanoscale zeolite pores and reported significant deviations from bulk liquid behavior^14^. Anderson et al. investigated interfacial water layers on silica surfaces using Raman spectroscopy and suggested the presence of an “ice-like” structural arrangement^15^.

More recently, interest has extended toward confined water at larger length scales approaching the submicron regime. Several groups employed infrared and Raman spectroscopy to study water in mesoporous silica and observed deviations in thermodynamic and physical properties relative to bulk liquid water^16–18^. Takehiko et al. examined water sealed in square silica microtubules using nuclear magnetic resonance (NMR) and proposed that molecular motion within the near-surface region (10–100 nm) was inhibited by abnormal proton transfer processes^19^. Le et al. investigated water confined in packings of glass beads with pore diameters ranging from 8 to 320 nm and found that the infrared spectra differed markedly from those of bulk water and varied systematically with pore size^20^. These effects were attributed to enhanced hydrogen bonding near the glass–water interface induced by geometric confinement.

It is noteworthy that existing studies on anomalous or solid-bound water in confined spaces exhibit two major limitations. First, most investigations rely predominantly on molecular dynamics simulations. Second, experimental and theoretical efforts have largely focused on nanoscale confinement. Consequently, the behavior of water confined in submicron-to-micron-scale geometries under ambient conditions remains poorly understood.

In this work, we employ a comprehensive set of experimental techniques to systematically investigate the morphology, mechanical behavior, and spectroscopic characteristics (Raman and infrared) of water confined in SiO₂ microtubules with submicron-to-micron inner diameters under normal conditions.

## Results

1. **Solid-like morphology and mechanical behavior of confined water**.

**Fig. 1:**
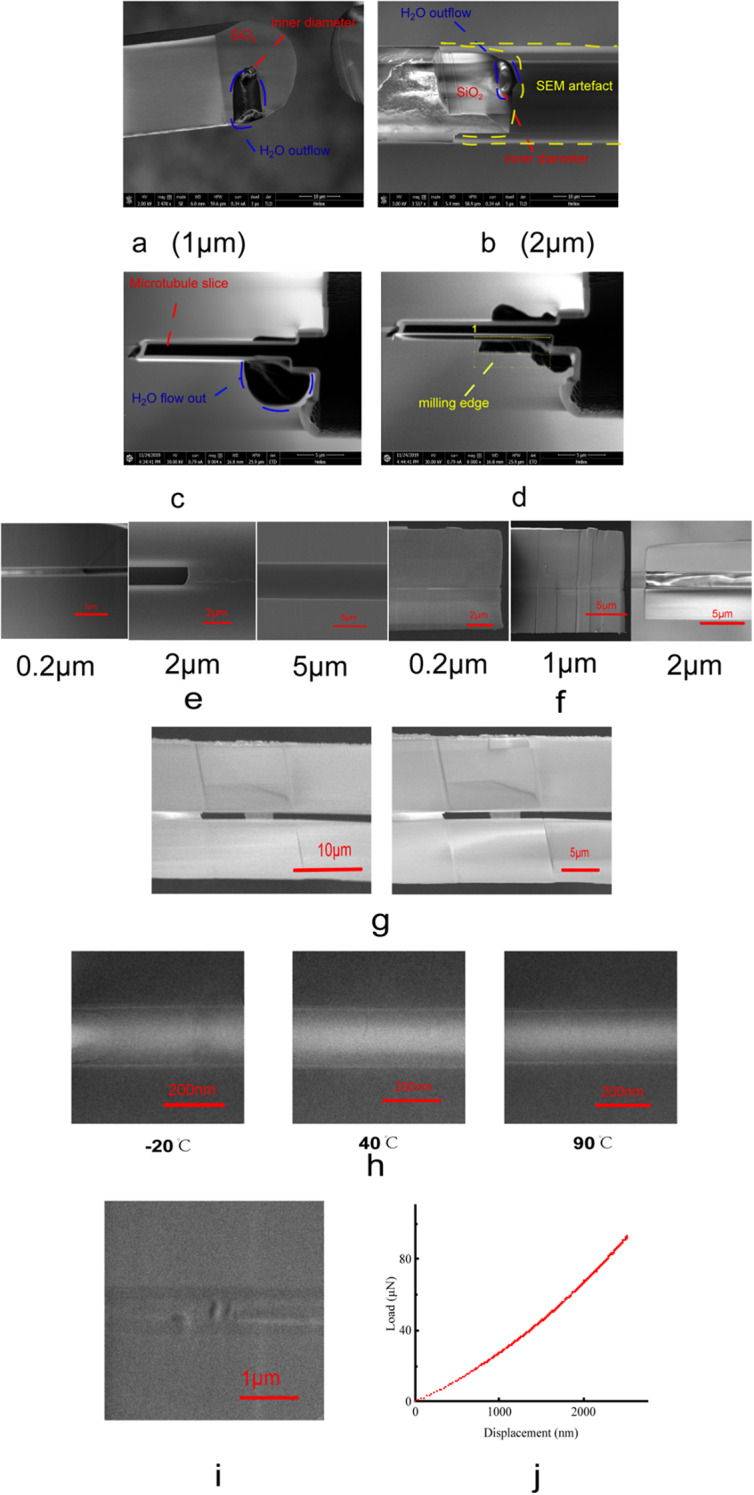
The morphology and mechanical properties of the SSW at 20°C and 10^− 5^Pa. (**a**, **b**) SEM images showing water extruded from SiO2 microtubules with inner diameters (ID) of 1 μm and 2 μm after cross-sectional cutting at 20 °C and 10− 5 Pa. (**c**, **d**) FIB-milled longitudinal sections of water confined in a SiO2 microtubule (ID = 2 μm), demonstrating shape retention after milling. (**e**) SEM images of cross sections of SiO2 microtubules with different inner diameters (0.2 μm, 2 μm, and 5 μm) after slicing. (**f**) SEM images of longitudinal slices of water confined in SiO2 microtubules with inner diameters of 0.2 μm, 1 μm, and 2 μm. (**g**) SEM images of a water slice (ID = 1 μm) immediately after preparation (left) and after 54 days of exposure to ambient conditions (right). (**h**) TEM images of water slices confined in SiO2 microtubules (ID = 0.4 μm) at − 20 °C, 40 °C, and 90 °C. (**i**) SEM image of nanoindentation marks on a water slice confined in a SiO2 microtubule (ID = 1 μm). (**j**) Force–displacement curve obtained from nanoindentation measurements of the confined water slice.

The submicron-to-micron-scale SiO₂-microtube-confined water (SSW) exhibits distinct solid-like morphological characteristics. As shown in Fig. 1a–d, water confined in SiO₂ microtubules with inner diameters of 1 μm and 2 μm can be shaped and sectioned while maintaining a stable form. Under SEM observation at 20 °C and a high vacuum of 10⁻⁵ Pa, focused ion beam (FIB) milling enabled direct sectioning of the confined water. The extruded material did not evaporate immediately but remained aggregated with a well-defined shape, indicating solid-like behavior (Supplementary Video 1–4). Additional SEM images showing confined water before and after FIB cutting are provided in Fig. S1.

Furthermore, the confined water could be milled into sharp-edged sections, similar to a solid material. Cross-sectional and longitudinal slicing of microtubules with different inner diameters revealed that water confined in microtubules ranging from 0.2 μm to 2 μm did not escape or evaporate under 20 °C and 10⁻⁵ Pa conditions (Fig. 1e, f). Additional SEM images of longitudinally milled microtubules with different inner diameters are provided in Fig. S2, and representative SEM images of confined water slices are shown in Fig. S3. These observations demonstrate that the confined water maintains a stable morphology under high vacuum and room temperature.

The morphology of the SSW is highly stable over both time and temperature. As shown in Fig. 1g, a water slice (100 nm in thickness) confined in a SiO₂ microtubule with an inner diameter of 1 μm remained intact after 54 days of exposure under ambient conditions and was still observed as a solid-like phase by transmission electron microscopy (TEM). Furthermore, temperature-dependent experiments conducted under high vacuum (10⁻⁵ Pa) demonstrate that the SSW maintains its solid-like morphology as the temperature increases from − 20 °C to 90 °C (Fig. 1h). Additional TEM images and corresponding electron diffraction patterns across this temperature range are provided in Fig. S4.

The mechanical response of the SSW is characteristic of a solid rather than a liquid. Nanoindentation combined with SEM was employed to probe the mechanical behavior of the confined water slices. As shown in Fig. 1i, the indentation probe produced well-defined and persistent imprints on the surface of the SSW slice, indicating resistance to deformation and the absence of fluid-like flow. The corresponding force–displacement curve (Fig. 1j) exhibits a monotonic increase in force with increasing displacement, which is typical of elastic–plastic deformation in solid materials (Supplementary Video 5).

In summary, both the morphological stability and the mechanical response of the SSW differ fundamentally from those of conventional liquid water and provide direct evidence for a solid-like confined water phase.

**Fig. 2:**
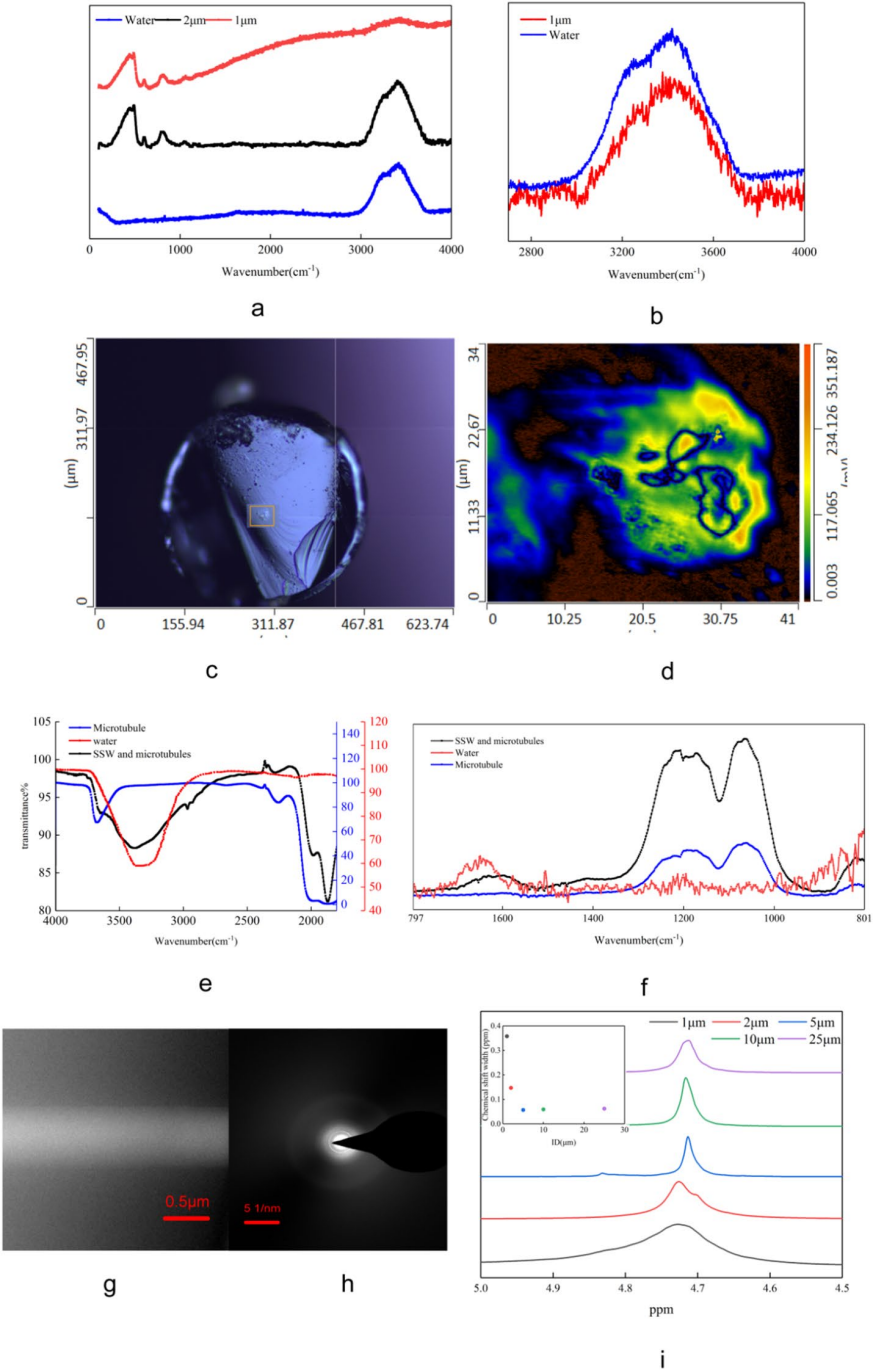
Structural characterization of the solid-like water (SSW). (**a**, **b**) Raman spectra of free water and water confined in SiO₂ microtubules with inner diameters of 2 μm and 1 μm under ambient conditions. (**c**) Cross-sectional infrared mapping of a radially cut SiO₂ microtubule (ID = 1 μm) filled with water. (**d**) Enlarged view of the region marked in (c), showing the spatial distribution of the infrared signal associated with confined water.(**e**) Mid-infrared spectra of free water, an empty SiO₂ microtubule (ID = 1 μm), and a water-filled microtubule (ID = 1 μm). (**f**) Near-infrared spectra of free water, an empty SiO₂ microtubule (ID = 1 μm), and confined water (SSW) in a microtubule (ID = 1 μm). (**g**, **h**) TEM image and corresponding electron diffraction pattern of a confined water slice in a SiO₂ microtubule with an inner diameter of 0.4 μm.(**i**) ¹H NMR spectra of water confined in SiO₂ microtubules with inner diameters of 25, 10, 5, 2, and 1 μm. Inset: dependence of the ¹H NMR linewidth on the microtubule inner diameter.

The Raman spectral features of the SSW differ markedly from those of bulk liquid water. Figure 2a presents the Raman spectra of water confined in SiO₂ microtubules with different inner diameters. For microtubules with an inner diameter of 2 μm, the Raman spectrum displays a typical broad band in the range of 3000–3600 cm⁻¹, corresponding to the O–H stretching vibrations of liquid water.

In contrast, the Raman spectrum of water confined in a SiO₂ microtubule with an inner diameter of 1 μm exhibits pronounced deviations (Fig. 2a, b), characterized by a substantially reduced peak intensity and a significantly broadened band profile. These spectral changes indicate a restricted molecular environment and suggest a substantial modification of the hydrogen-bonding network of the confined water relative to the bulk phase.

To confirm the spatial origin of the infrared response, we performed cross-sectional IR mapping on a radially cut, water-filled SiO₂ microtubule (ID = 1 μm). The map was constructed from the integrated absorbance over 1500–1750 cm⁻¹, which captures the H–O–H bending region while avoiding the dominant Si–O–Si framework bands at 1100–1200 cm⁻¹. As shown in Fig. 2c, the mapped signal is predominantly localized within the microtubule lumen rather than the surrounding SiO₂ matrix. The enlarged view in Fig. 2d further highlights the concentrated distribution within the selected region of interest (color scale: low to high intensity). Based on this spatial verification, Fig. 2e, f compare the IR spectra of bulk water, an empty SiO₂ microtubule (ID = 1 μm), and a water-filled microtubule (ID = 1 μm) (see Additional Fig. 5 for other sizes). For the empty microtubule, characteristic absorption bands are observed at 1100–1200 cm⁻¹^24^, corresponding to Si–O–Si framework vibrations, together with a distinct band at ~ 3670 cm⁻¹^25–27^ associated with surface silanol (SiOH) groups.

Compared with empty microtubules of larger inner diameters (e.g., 2 μm; see Fig. S5), the silanol-related band at ~ 3670 cm⁻¹ is markedly stronger for the 1 μm microtubule, indicating a significantly higher density of surface silanol groups in the smaller microtubule.

After subtracting the characteristic absorption bands of the empty SiO₂ microtubule from the IR spectrum of the water-filled microtubule (ID = 1 μm), a distinct absorption band remains in the range of 2900–3000 cm⁻¹, which differs from that of conventional liquid water (Fig. 2e, f). A corresponding feature is also observed in the Raman spectra (see Fig. S6), supporting that this band originates from the confined water rather than the SiO₂ microtubule matrix.

In addition, the IR band associated with the H–O–H bending vibration of the confined water exhibits a red shift of approximately 50 cm⁻¹ relative to bulk liquid water (centered at ~ 1640 cm⁻¹). This red shift suggests an altered hydrogen-bonding environment and modified vibrational dynamics under submicron confinement.

Figure 2e shows that the mid-infrared spectral features of water confined in a SiO₂ microtubule with an inner diameter of 1 μm are mainly distributed in the range of 2700–3700 cm⁻¹. Although the absorption band associated with surface silanol groups (~ 3670 cm⁻¹) partially overlaps with this region (Fig. 2e), the O–H stretching band of the confined water is clearly broader than that of conventional liquid water. This band broadening indicates an alteration of the molecular stretching vibration behavior of the confined water relative to the bulk phase.

The ¹H NMR spectral features of the SSW also differ from those of bulk liquid water. Figure 2i presents the ¹H NMR spectra of water confined in SiO₂ microtubules with different inner diameters. For microtubules with inner diameters of 25 μm, 10 μm, and 5 μm, the resonance peak appears at approximately 4.7 ppm with similar linewidths, which is characteristic of conventional liquid water.

In contrast, water confined in SiO₂ microtubules with inner diameters of 2 μm and 1 μm exhibits pronounced deviations (Fig. 2i), characterized by substantial line broadening and a slight downfield shift of the resonance peak. Both the linewidth expansion and the chemical shift increase are more pronounced for the 1 μm microtubule than for the 2 μm microtubule, indicating progressively restricted molecular mobility and enhanced hydrogen bonding with decreasing microtubule diameter.

The electron diffraction pattern of the SSW does not exhibit features characteristic of crystalline ice. As shown in Fig. 2g, h, the diffraction pattern of the SSW slice obtained by TEM is neither composed of discrete spots typical of a single crystal nor of sharp concentric rings characteristic of a polycrystalline phase. Instead, it presents a diffuse halo, which is indicative of an amorphous structure.

This result suggests that, although the confined water exhibits solid-like morphological and mechanical behavior, it lacks long-range crystalline order. While the detailed molecular arrangement of the SSW cannot be resolved from the present diffraction data, the observed diffuse scattering confirms that the solid-like water phase is structurally amorphous (see Fig. S7 for further results).

**Fig. 3:**
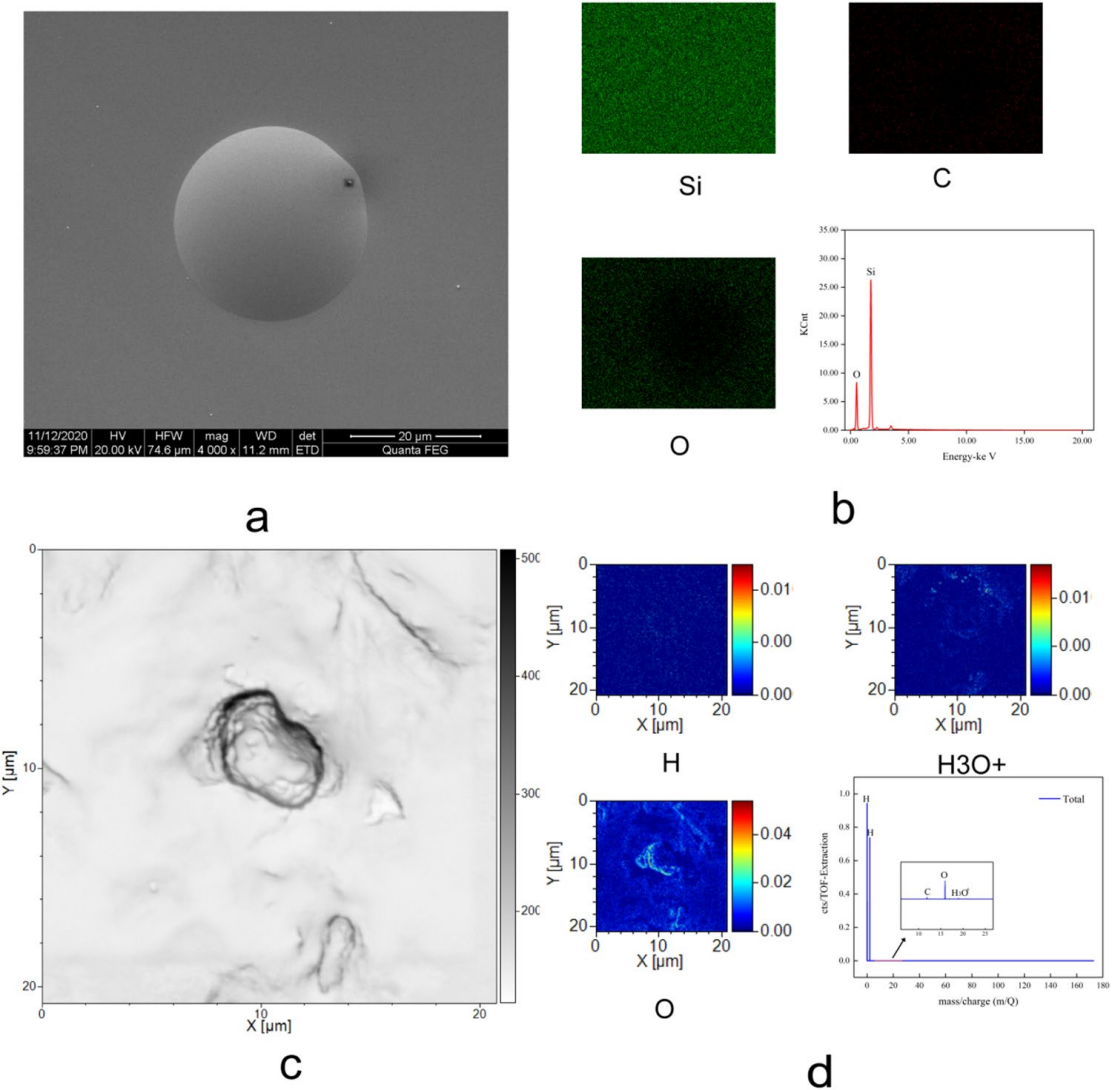
Elemental and mass-spectrometric characterization of the solid-like water (SSW) extruded from a SiO₂ microtubule (ID = 1 μm). (**a**) SEM image of the extruded SSW from a SiO₂ microtubule (ID = 1 μm).(**b**) EDS elemental maps (Si, C, and O) and the corresponding energy spectrum acquired from the same region as in (**a**).(**c**) SEM image of the extruded SSW obtained using SEM–FIB–ToF-SIMS after storage under partial vacuum for two weeks.(**d**) ToF-SIMS elemental maps of H, H₃O⁺, and O acquired from the same region as in (**c**), together with the corresponding mass spectrum. The elemental maps indicate that the extruded material consists predominantly of hydrogen- and oxygen-containing species.

2. **The solid-like water is not attributable to extrinsic impurities**.

- The elemental composition of the material extruded from SiO₂ microtubules with an inner diameter of 1 μm was examined by energy-dispersive X-ray spectroscopy (EDS) and time-of-flight secondary ion mass spectrometry (ToF-SIMS) (Fig. 3a–d; see Fig. S8 for supporting results). The EDS spectrum (Fig. 3b) shows only oxygen and silicon signals, consistent with contributions from the SiO₂ microtubule matrix and water-derived oxygen, and reveals no detectable peaks from other elements.
- The ToF-SIMS spectra of the extruded material (Fig. 3c, d) further indicate that the dominant species are hydrogen- and oxygen-containing ions. In addition to these components, a weak carbon signal is observed, which is attributed to trace surface contamination arising from exposure to ambient air (see Fig. S9). A peak at mass-to-charge ratio m/z = 19 is also detected and is assigned to the hydronium ion (H₃O⁺) rather than fluorine, because no fluorine signal is observed in the EDS measurements. The presence of H₃O⁺ is plausibly associated with proton transfer involving water molecules and deprotonated silanol groups on the inner surface of the SiO₂microtubules.
- In summary, the combined EDS and ToF-SIMS analyses show no evidence of foreign elemental impurities in the extruded material. These results support that the observed solid-like phase is an intrinsic form of confined water formed within the SiO₂ microtubules, rather than a product of contaminating substances.

- **3. Exploration of Critical Factors Governing the Properties of SSW**.
  1. Influence of the Inner Surface Chemistry of SiO₂ Microtubules

### **High-density aggregation of surface silanol groups**

**Fig. 4:**
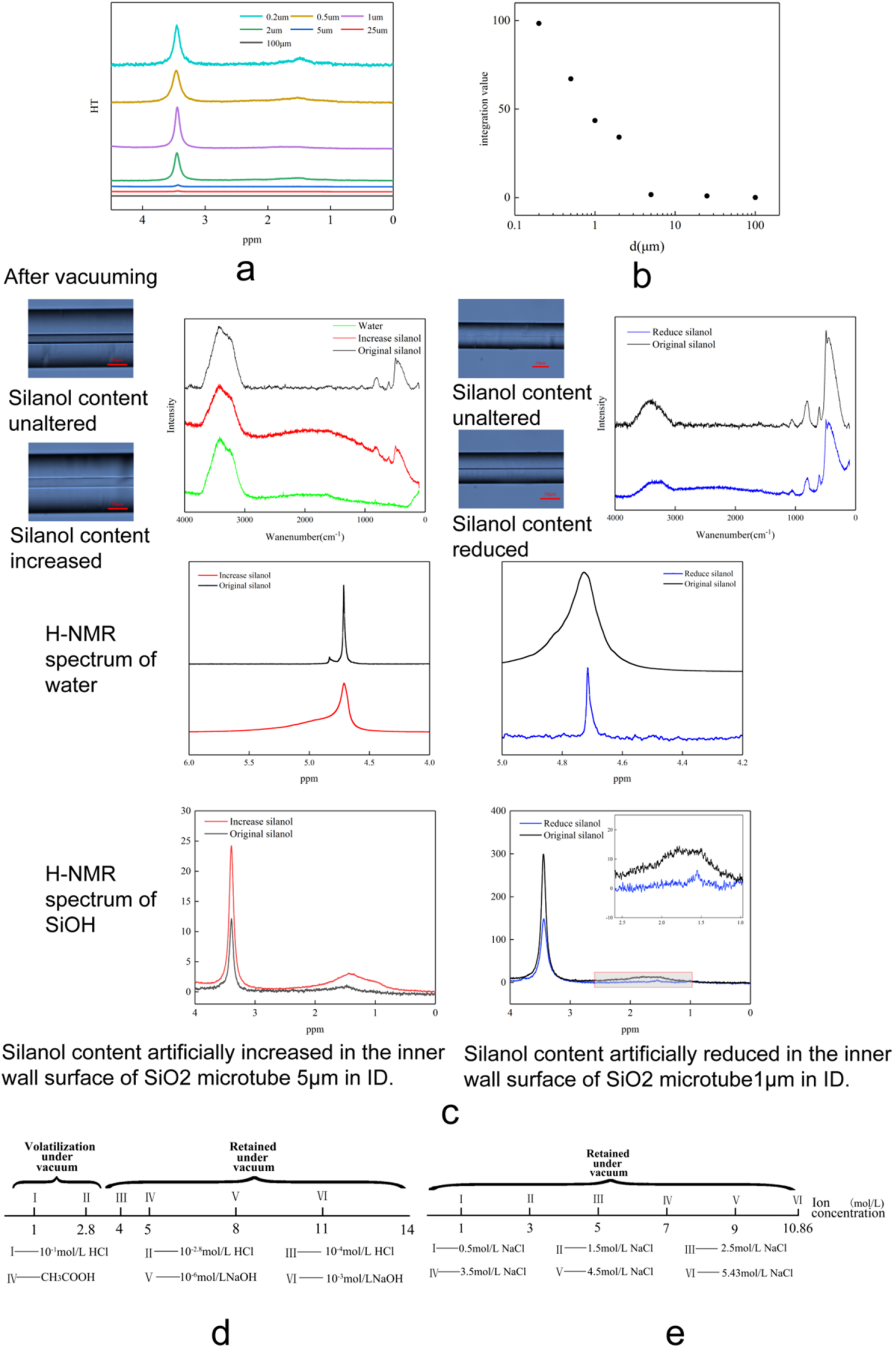
Effects of surface silanol density, pH, and ionic strength on the properties of confined water. (**a**, **b**) Quantification of surface silanol groups: (**a**) ¹H NMR spectra of SiO₂ microtubules with different inner diameters (IDs) and (**b**) the corresponding integrated peak areas plotted as a function of microtubule ID.(**c**) Reversible phase transitions induced by surface modification. Microtubules with an ID of 5 μm were treated with H₂O₂ to increase the surface SiOH density, whereas microtubules with an ID of 1 μm were treated with NaOH to decrease it. From top to bottom: optical images after vacuum exposure, Raman spectra, and ¹H NMR spectra before and after treatment. (Green curves: pure water; black curves: pre-treatment; colored curves: post-treatment.) (**d**, **e**) Optical observations of water confined in a 1 μm microtubule after injection of (**d**) aqueous solutions with different pH values and (**e**) NaCl solutions with different ionic concentrations, followed by vacuum exposure for 10 min.

The SiO₂ microtubules exhibit an unusually high density of silanol (SiOH) groups. The ¹H NMR spectra of microtubules with seven different inner diameters (IDs) were acquired to quantify the SiOH content. As shown in Fig. 4a, two characteristic resonance peaks appear at approximately 3.4 and 1.5 ppm. According to previous studies^21,23,26,28–30^, these peaks correspond to SiOH protons, and their integrated intensities serve as a quantitative measure of the relative silanol abundance.

Figure 4a and b reveal a pronounced size dependence of the SiOH content. When the inner diameter decreases into the range of 2–5 μm, the SiOH content in the microtubule matrix increases sharply. In contrast, for microtubules with IDs larger than 5 μm, the SiOH content remains at a negligible level. Quantitatively, the SiOH content in microtubules with IDs of 0.2 μm and 1.0 μm is approximately 2098 and 927 times higher, respectively, than that in 100 μm microtubules. These results demonstrate a dramatic enrichment of SiOH groups in submicron-scale microtubules, coinciding precisely with the critical diameter range where solid-like water is observed.

To probe the spatial distribution of these silanol groups, a saturated NaOH solution was injected into a 1 μm microtubule to selectively etch the inner surface. XPS depth profiling indicates a reaction depth of approximately 5 nm (Fig. S9). The SiOH content before and after NaOH treatment was quantified by ¹H NMR spectroscopy. As shown in Fig. 4c and Fig. S10, the integrated intensity of the SiOH signal decreased from 52.92 (untreated) to 32.77 (NaOH-treated), corresponding to a reduction of approximately one-third.

This result implies that at least one-third of the total SiOH content in the 1 μm microtubule is concentrated within an ultra-thin interfacial layer (~ 5 nm) at the inner wall. Based on this geometry, the effective density of SiOH groups in this interfacial region is estimated to be on the order of 10⁶ times higher than that in the outer bulk region of the matrix (Fig. S12).

Compared with 100 μm microtubules, the SiOH distribution in 1 μm microtubules is markedly heterogeneous, with strong enrichment near the inner surface (Fig. S10). To visualize this, a 1 μm microtubule was longitudinally milled by FIB to expose its interior, and hydrogen mapping was performed using dual-beam SEM–mass spectrometry. The hydrogen signal—a proxy for silanol groups—was significantly more intense near the inner surface than in the outer matrix (Fig. S11). These findings provide direct experimental evidence for the dense aggregation of SiOH groups at the inner wall of submicron SiO₂ microtubules.

### **Modulation of surface SiOH density and reversible phase transitions**

To establish causality, surface modification experiments were conducted. Treatment with saturated NaOH solution reduced the surface SiOH density of the 1 μm microtubules (Fig. 4c). Following this treatment, the previously solid-like confined water reverted to a conventional liquid-like state. This transition was accompanied by the recovery of bulk-like Raman and ¹H NMR spectral features, indicating that the anomalous solid-like behavior is suppressed when the interfacial silanol density is diminished.

Conversely, when 5 μm microtubules (which originally contained liquid water) were treated with hydrogen peroxide solution to artificially increase the surface silanol density, the confined water transformed from a normal liquid state into the solid-like state (Fig. 4c). This transition was marked by the emergence of broadened and shifted ¹H NMR signals characteristic of the SSW phase.

These reversible phase transitions demonstrate that the formation of the solid-like water phase is critically sensitive to the density of surface silanol groups, establishing surface chemistry as the dominant factor governing the phase behavior of water confined in SiO₂ microtubules.

2. Influence of pH on the Stability of SSW

The stability of the SSW was systematically examined over a pH range of 1 to 11. As shown in Fig. 4d, at ambient temperature, aqueous solutions with pH values between 2.8 and 11 confined in 1 μm microtubules exhibited a stable solid-like state. Even after prolonged exposure to high vacuum, these samples showed no signs of evaporation (Fig. S13).

In contrast, when the pH was reduced to the strongly acidic range (pH = 1–2.8), the confined water volatilized completely under high vacuum, indicating a reversion to conventional liquid-like behavior (Fig. S13).

These results indicate that the solid-like state is strongly pH-dependent. Extremely acidic conditions destabilize the solid-like phase, whereas near-neutral and weakly alkaline conditions favor its formation. This behavior aligns with the protonation equilibrium of surface silanol groups: at very low pH, the suppression of silanol deprotonation likely disrupts the interfacial hydrogen-bonding network required to maintain the solid-like structure.

(3)  Influence of Ionic Strength

The effect of ionic concentration was examined by introducing aqueous NaCl solutions (0–15 mol/L) into 1 μm microtubules. As shown in Fig. 4e, all samples within this concentration range exhibited a solid-like state at ambient temperature. Even after prolonged vacuum exposure, the confined solutions remained stable (Fig. S14).

These results demonstrate that ionic strength has negligible influence on the formation and stability of the solid-like phase. This suggests that the solid-like behavior is primarily governed by short-range interfacial interactions between water molecules and surface silanol groups, rather than by bulk ionic effects or variations in Debye screening length.

## Discussion

Solid-like water confined in submicron-to-micron SiO_2_ microtubules is a verifiable phenomenon under ambient conditions. This conclusion is supported by a large body of reproducible experimental evidence obtained using multiple independent characterization techniques. Unlike previously reported confined water phases, this water exhibits an unusually stable solid-like morphology under normal to elevated temperatures (25 °C to 90 °C) and low to ambient pressures (10^− 5^Pa to 1.01 × 10^5^Pa).

The material displays solid-like processing behavior, as it can be shaped by focused ion beam cutting. It also shows solid-like mechanical responses, deforming under applied stress rather than flowing as a conventional liquid. Furthermore, Raman, infrared, and ¹H NMR measurements reveal distinct spectral features compared with bulk liquid water, indicating a substantial reduction in the molecular kinetic energy of this confined water phase.

What distinguishes the molecular structure of this solid-like water from that of bulk liquid water? Compared with conventional liquid water, the infrared spectral band associated with molecular bending vibrations exhibits a pronounced red shift, and the ¹H NMR resonance shows a substantially broadened linewidth, nearly an order of magnitude larger than that of bulk water. These spectral features indicate strongly suppressed molecular mobility and a modified hydrogen-bonding environment. The observed red shift of the bending vibration band is consistent with strengthened hydrogen bonding, which modifies the local potential energy surface of the water molecules and lowers the corresponding vibrational frequency.

Although the electron diffraction pattern of the solid-like water (ID ≤ 1 μm) remains diffuse and lacks any signature of long-range crystalline order, the combined spectroscopic evidence consistently demonstrates that the molecular kinetic energy of this confined water phase is significantly lower than that of conventional liquid water. Given the complexity of the submicron interface, future studies incorporating molecular dynamics (MD) simulations will be critical to resolving the precise molecular arrangement and hydrogen-bond network details that currently remain elusive.

The transition from conventional liquid water to solid-like water occurs at a characteristic length scale.

Macroscopically, a critical inner diameter (ID) of approximately 2–5 μm is observed, below which the morphology of confined water transforms from a liquid-like to a solid-like state (Fig. 1e, f and Fig.S2). Microscopically, the linewidth of the ¹H NMR resonance, which reflects molecular kinetic energy, increases sharply as the microtubule ID decreases within this same range (Fig. 2i). In parallel, the silanol (SiOH) density in the SiO_2_ microtubule matrix also rises steeply when the ID falls below 2–5 μm. Notably, the onset of rapid SiOH enrichment coincides with the emergence of distinct water morphology and reduced molecular mobility.

This correspondence is unlikely to be accidental. As the inner diameter decreases, the density of silanol groups on the interior surface increases markedly (Fig. 4c and Fig.S12), strengthening interfacial hydrogen bonding and restricting molecular motion of the confined water. These interfacial hydrogen bonds act as anchoring points that stabilize the local hydrogen-bond network and suppress both translational and rotational dynamics of adjacent water molecules. When the molecular kinetic energy is reduced to a sufficiently low metastable level, the confined water undergoes a transition to a solid-like state, exhibiting unusual macroscopic morphology and solid-like mechanical behavior (Fig. 1). From a physical perspective, the increase in surface silanol density effectively deepens the interfacial potential well experienced by water molecules and raises the energetic barrier for translational and rotational motion, thereby stabilizing a low-mobility, solid-like state under submicron confinement.

Comparatively, water phase transitions under nanoscale confinement (< 10 nm) are typically governed by strong geometric restriction of the entire confined volume. In contrast, the phenomenon observed here in submicron microtubules (2–5 μm) points to a distinct mechanism dominated by interfacial chemistry: the exceptionally high density of surface silanol groups may induce an extended interfacial ordering effect that propagates substantially farther from the wall than in conventional nanoscale systems. This feature differentiates the present submicron confinement regime from purely geometry-driven nanoscale confinement.

It should also be noted that the present experiments were conducted using microtubes with circular cross-sections. The influence of confinement geometry, such as slit-like channels or non-cylindrical pore shapes, on the formation and stability of the solid-like water phase remains to be explored and represents an important direction for future work.

The discovery of a solid-like water phase that remains stable under ambient conditions is expected to stimulate further investigation of confined water behavior in submicron-to-micron-scale systems. This finding also suggests potential opportunities for exploiting such a phase in a range of technological contexts.

Possible applications include micro- and nanoreactors or catalysts for chemical synthesis, where an immobilized water network may help stabilize reactive intermediates; enhanced fluidic control in micro-machines, in which the solid-like phase could function as a passive valve without external energy input; regulation of mass and heat transfer in micro- and nanoporous media; and non-freezing alternatives for biological sample preparation in life-science applications, offering a route to preserve cellular integrity by suppressing the formation of sharp crystalline ice.

In addition, this phenomenon may have implications for fluid transport in tight geological formations, where high silanol densities and submicron pore structures are ubiquitous. The formation of a solid-like confined water phase under such conditions could provide a microscopic perspective on anomalous flow resistance and abnormally low permeability observed in silica-rich tight porous media. These prospects highlight the broader relevance of solid-like confined water beyond fundamental studies of interfacial and confined fluids. It should be noted that the current stability tests were conducted over a period of 54 days, which is sufficient to demonstrate that this phase is not a transient state under ambient conditions. However, longer-term stability assessments over extended timescales will be required to fully evaluate its suitability for practical applications.

In summary, the identification of a solid-like water phase in submicron-to-micron SiO_2_ microtubules raises several fundamental questions that merit further investigation. These include the physicochemical origin of this phase, the detailed molecular arrangement and hydrogen-bond network within it, the spatial heterogeneity of its structure and properties across the confined geometry, and the dynamic process by which liquid water transforms into the solid-like state, including the kinetics and possible intermediate states.

Beyond water and silica, it also remains to be explored whether other fluids—such as alkanes, alcohols, gases, or multicomponent mixtures—may undergo analogous solid-like transitions under similar submicron confinement conditions or in the presence of different wall materials and surface chemistries. Addressing these questions will be essential for establishing a comprehensive framework for phase behavior in interfacially dominated confined systems.

## Conclusions

In summary, we report the discovery of a stable solid-like water phase confined within submicron-to-micron-scale SiO₂ microtubules under ambient conditions. This confined water exhibits clear solid-state characteristics, including sectioning by focused ion beam (FIB) milling, plastic deformation under mechanical loading, and remarkable stability over a wide temperature range (− 20 to 90 °C) and pressure range from high vacuum to atmospheric pressure. In contrast to previously reported confined ice or interfacial water structures that require sub-10 nm confinement or cryogenic conditions, the solid-like phase observed here persists at the mesoscale.

Systematic experiments demonstrate that an exceptionally high density of surface silanol (SiOH) groups on the inner walls of the microtubules is the key factor governing the formation of this phase. The resulting interfacial hydrogen-bond network imposes long-range ordering on the confined water, strongly suppressing molecular mobility even in relatively large pores. This finding extends the conventional length scale of confinement-induced phase transitions and emphasizes the dominant role of interfacial chemistry in controlling the physical state of fluids at the mesoscale.

These results provide new insight into the behavior of water in submicron porous systems, such as tight geological formations and biological microenvironments. Moreover, the reversible control of this solid-like phase through surface chemical modification suggests potential opportunities for applications in nanofluidic regulation, energy storage and conversion, and the design of solid-state or hybrid electrolytes.

### Method

#### Preparation of samples for dual-beam scanning electron microscopy

SiO₂ microtubules (PolyMicro Inc., USA) were first immersed in hydrofluoric acid to remove the polyimide coating and to reduce the wall thickness. The microtubules were then thoroughly rinsed with ultrapure water and dried in an oven. After drying, ultrapure water was injected into the thinned microtubules.

The filled microtubules were placed onto single-slit pure copper TEM grids and fixed perpendicular to the slit using fast-curing adhesive. To improve electrical conductivity, a thin gold layer was deposited on the samples. The regions of the microtubules suspended over the slit were subsequently processed using a dual-beam scanning electron microscope (FIB-SEM) to prepare cross-sectional slices for transmission electron microscopy observation.

## Preparation of samples for Raman, infrared spectroscopy, mass spectrometry, and energy-dispersive spectroscopy

Two types of samples were prepared: whole-tube samples and end-face samples.

For whole-tube samples, thinned microtubules were filled with ultrapure water using a high-pressure syringe pump. Both ends of the microtubules were then sealed with UV-curable adhesive to prevent leakage.

For end-face samples, ultrapure water was continuously injected into the microtubules using a syringe pump, while the tube end was positioned inside a sealed chamber made of PEEK material. The confined chamber was evacuated using a vacuum pump. After continuous injection for 12 h, the samples were removed and transferred to the vacuum chamber for subsequent spectroscopic and analytical measurements.

### NMR sample preparation method

SiO₂ microtubules were cut into 14 cm long segments. For each measurement, ten microtubules with identical inner diameters were used. The microtubules were first immersed in hydrofluoric acid to remove the polymer coating, thoroughly rinsed with purified water, and dried in an oven.

For measurements of silanol (SiOH) content, the dried microtubules were directly used as solid samples. For measurements of confined water, the treated microtubules were subsequently filled with ultrapure water.

### Surface modification for silanol density control

To modify the surface silanol density, the microtubules were first immersed in hydrofluoric acid to remove the coating and then rinsed with ultrapure water. Subsequently, 30 wt% hydrogen peroxide solution or saturated sodium hydroxide solution was injected into the microtubules. The samples were heated in an oven at 60 °C for 8 h. After treatment, the microtubules were rinsed and then filled with ultrapure water for subsequent measurements.

### Preparation of samples with different aqueous solutions

After coating removal by hydrofluoric acid and cleaning with ultrapure water, the microtubules were filled with various aqueous solutions using a high-pressure syringe pump (Teledyne Isco D) under a pressure difference of 8 MPa. The injected solutions included acetic acid, concentrated hydrochloric acid, dilute hydrochloric acid, sodium bicarbonate solution, sodium carbonate solution, and sodium chloride solutions with different ionic concentrations.

### Vacuum test

The prepared samples were placed in a vacuum chamber with a pressure of 2 Pa for at least 10 min. After vacuum treatment, the samples were removed and immediately examined under ambient conditions using an optical microscope.

### Characterization and instrumentation

Raman spectroscopy and correlative Raman–electron microscopy were performed using a TESCAN RISE microscope and a confocal Raman microscope (Renishaw inVia Qontor). Infrared microspectroscopy was carried out using a micro-imaging FTIR spectrometer (Thermo Scientific iN10 MX). Mass spectrometry and elemental mapping were conducted with a FIB–TOF-SIMS system (GAIA3) and a dual-beam scanning electron microscope (FEI Helios NanoLab 600i).

Morphological observation and mechanical characterization were performed using a scanning electron microscope combined with a nanoindenter (Hysitron PI88). Nuclear magnetic resonance measurements were carried out using a 700 MHz NMR spectrometer (Bruker AVANCE NEO). Transmission electron microscopy (TEM) and selected-area electron diffraction (SAED) were performed using a 200 kV field-emission TEM (FEI Tecnai G2 F20) equipped with a cryogenic sample holder.

X-ray photoelectron spectroscopy (XPS) measurements were conducted using a Kratos AXIS ULTRA DLD spectrometer. Nanoscale infrared and Raman measurements were performed using a non-contact submicron-resolution photothermal infrared (O-PTIR) system (Photothermal Spectroscopy Corp., mIRage). Optical microscopy was used for routine observation of sample morphology.

## Supplementary Information

Below is the link to the electronic supplementary material.

Supplementary Material 1

## Data Availability

The datasets generated and/or analyzed during the current study are available from the corresponding author upon reasonable request.
