# Supplementary material for: A stable solid-like water at normal condition
Source: Sci Rep. 2026 May 7;16:14588. doi: 10.1038/s41598-026-42682-x (PMC13153236; doi:10.1038/s41598-026-42682-x)
Supplement: Supplementary file 1 — Supplementary Material 1 [file 41598_2026_42682_MOESM1_ESM.docx]

**Supplementary Information**

**A stable solid-like water at normal condition**

An Wei-qing^4^, Yue Xiang-an* ^1,2^, Zou Ji-rui*^1,3^

1. State Key Laboratory of Petroleum Resources and Prospecting, China University of Petroleum (Beijing), Beijing 102249, China.

2. College of Petroleum Engineering, China University of Petroleum-Beijing, Beijing 102249, China.

3. College of Energy Innovation, China University of Petroleum-Beijing, Beijing 102249, China.

4. Research lnstitute Exploration & Development of Xinjiang Oilfield Company, CNPC, Karamay, Xinjiang.China.

*Corresponding Author at: China University of Petroleum (Beijing), China. E-mail address: [yxa@cup.edu.cn;](mailto:yxa@cup.edu.cn;) zou_jirui@cup.edu.cn

**Supplementary Figures**

**Figure S1**


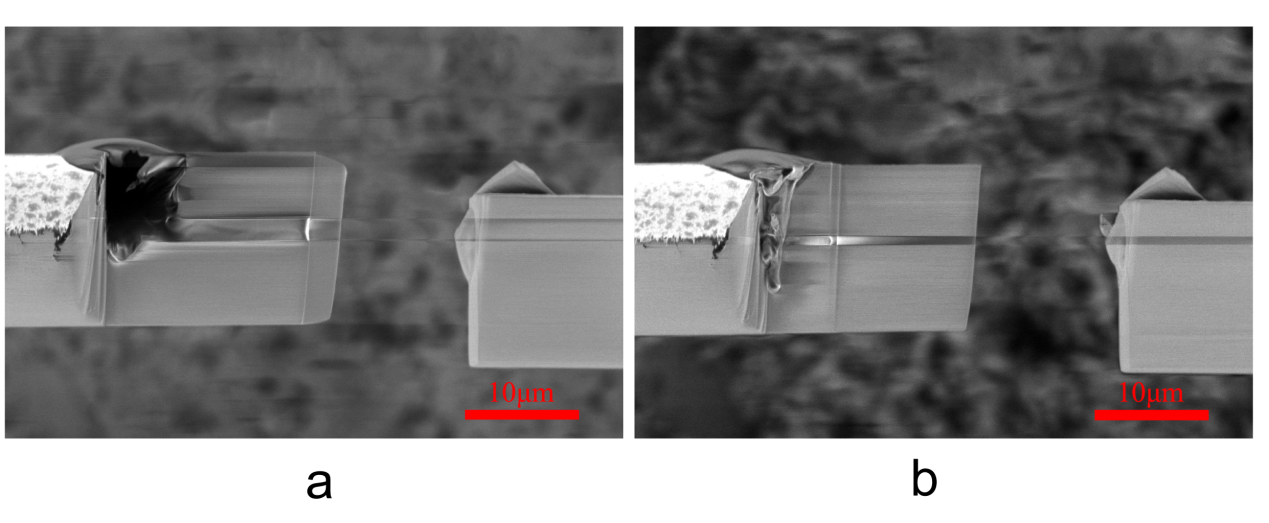


**Figure S1. SEM images of confined water before and after FIB cutting.**

SEM images of water confined in a SiO₂ microtubule (inner diameter = 2 μm) before (a) and after (b) focused ion beam (FIB) cutting (20 °C, 10⁻⁵ Pa). Scale bars: 10 μm.

**Figure S2**


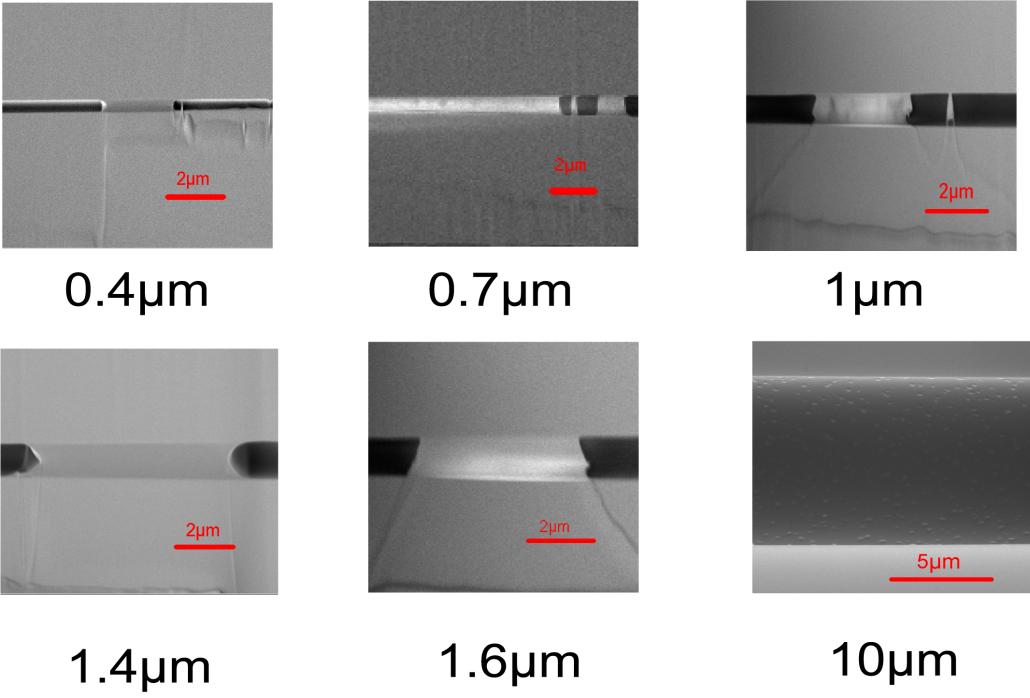


**Figure S2. SEM images of longitudinally milled SiO₂ microtubules with different inner diameters.**

SEM images of SiO₂ microtubules (inner diameters: 0.4, 0.7, 1.0, 1.4, 1.6, and 10 μm) after longitudinal focused ion beam (FIB) milling, acquired at 20 °C under high vacuum (10⁻⁵ Pa). Dark regions correspond to empty space, whereas bright regions indicate the confined water (SSW).

**Figure S3**


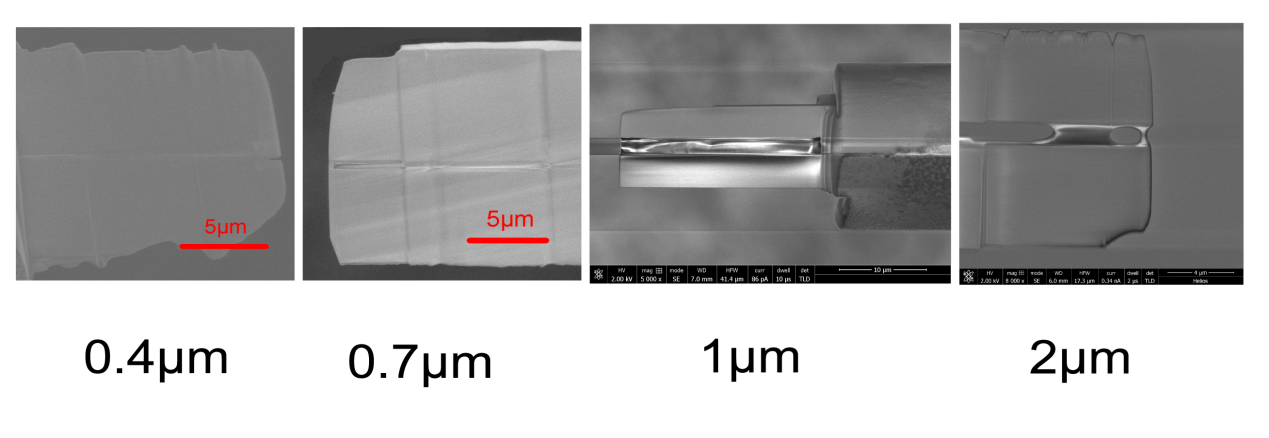


**Figure S3. SEM images of confined water slices in SiO₂ microtubules with different inner diameters.**

SEM images of confined water slices in SiO₂ microtubules (inner diameters: 0.4, 0.7, 1.0, and 2.0 μm), obtained at 20 °C under high vacuum (10⁻⁵ Pa). Dark gray regions correspond to empty space, whereas light-gray to white regions indicate the confined water (SSW).

**Figure S4**


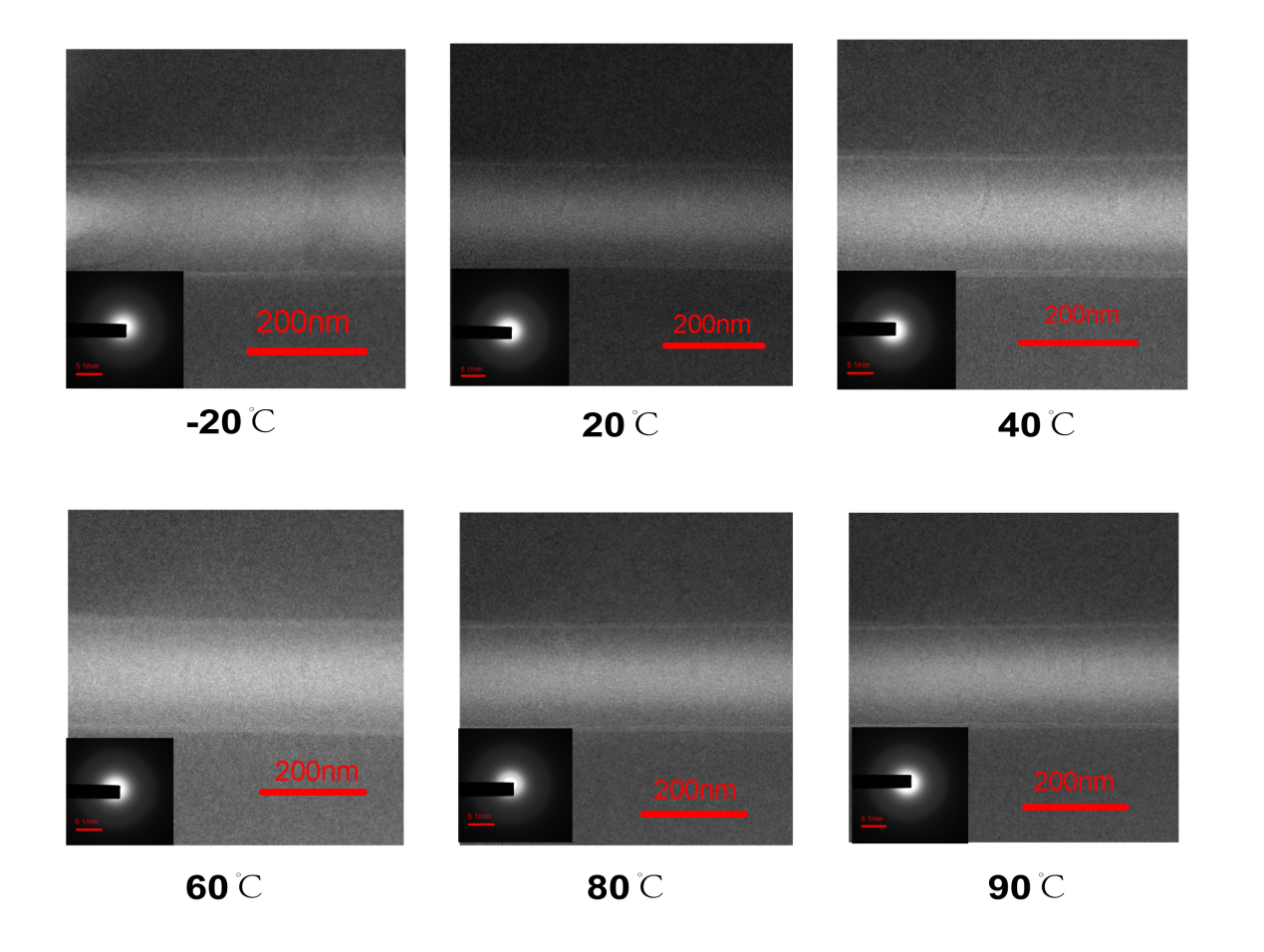


**Figure S4. TEM images and electron diffraction patterns of confined water at different temperatures.**

TEM images of water slices confined in a SiO₂ microtubule (inner diameter = 0.4 μm) acquired at temperatures ranging from -20 °C to 90 °C. The SiO₂ microtubule walls appear as parallel dark-gray bands at the top and bottom of each image, whereas the confined water is observed as a lighter-gray band between them. Corresponding electron diffraction patterns are shown in the lower-left corners of the images.

**Figure S5**


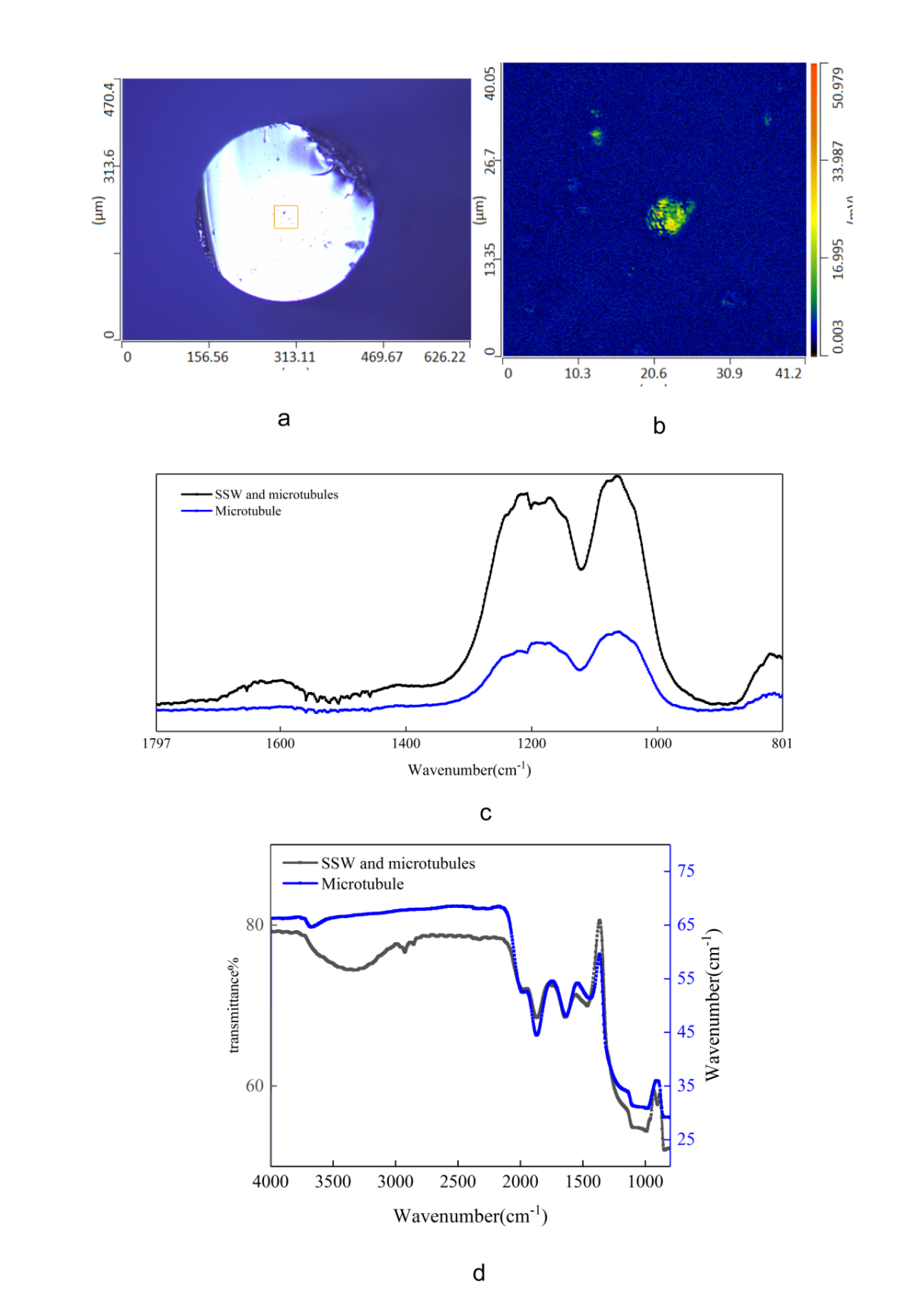


**Figure S5. Infrared characterization of water confined in SiO₂ microtubules with different inner diameters.**

1. Optical micrograph of a water-filled SiO₂ microtubule (ID = 0.5 μm) recorded during micro-area infrared measurements. (b) Enlarged infrared mapping of the region indicated in (a), showing the spatial distribution of the infrared signal across the microtubule cross-section. (c) Near-infrared spectra of an empty SiO₂ microtubule (ID = 0.5 μm) and the same microtubule after water injection. (d) Mid-infrared spectrum of a water-filled SiO₂ microtubule with an inner diameter of 2 μm.

**Figure S6**


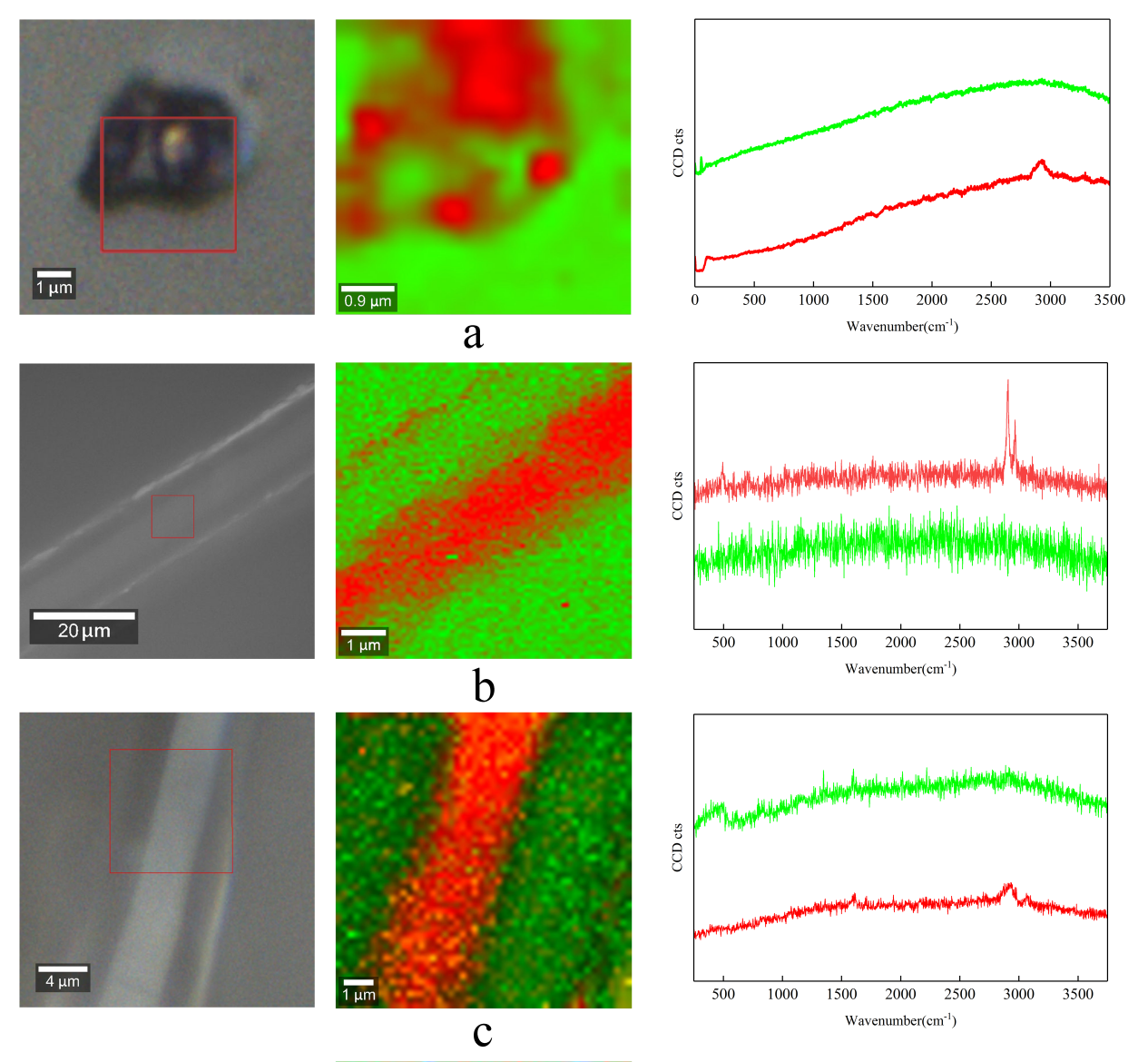


**Figure S6. Raman characterization of the interfacial region between confined water and the inner wall of SiO₂ microtubules with different inner diameters.**

(a–c) Raman measurements at the water–wall interface in SiO₂ microtubules with inner diameters of 0.5 μm, 1 μm, and 2 μm, respectively. For each panel, the left image shows the corresponding SEM micrograph, the middle image shows the Raman areal map, and the right panel shows Raman spectra extracted from representative regions. Green curves correspond to spectra acquired from the microtubule wall, whereas red curves represent spectra obtained from the interfacial region between the confined water and the inner wall.

**Figure S7**


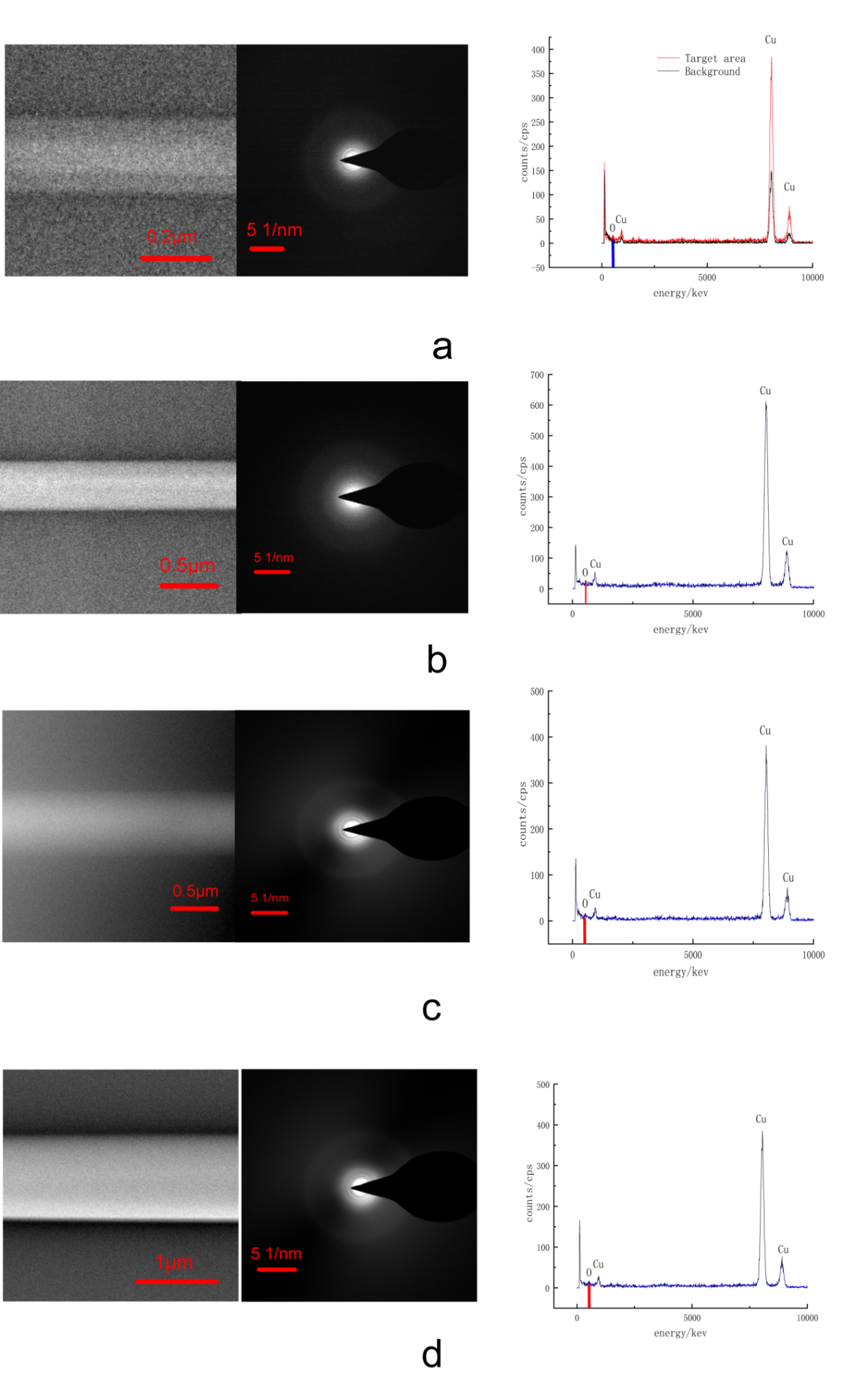


**Figure S7. TEM, SAED, and EDS characterization of confined water (SSW) in SiO₂ microtubules.**

(a–d) TEM images (left), selected-area electron diffraction (SAED) patterns (middle), and EDS spectra (right) of water slices confined in SiO₂ microtubules with inner diameters of 0.2, 0.4, 0.7, and 1 μm, respectively. The EDS spectra acquired from the confined-water regions show only oxygen signals, with no detectable solid impurities.

**Figure S8**


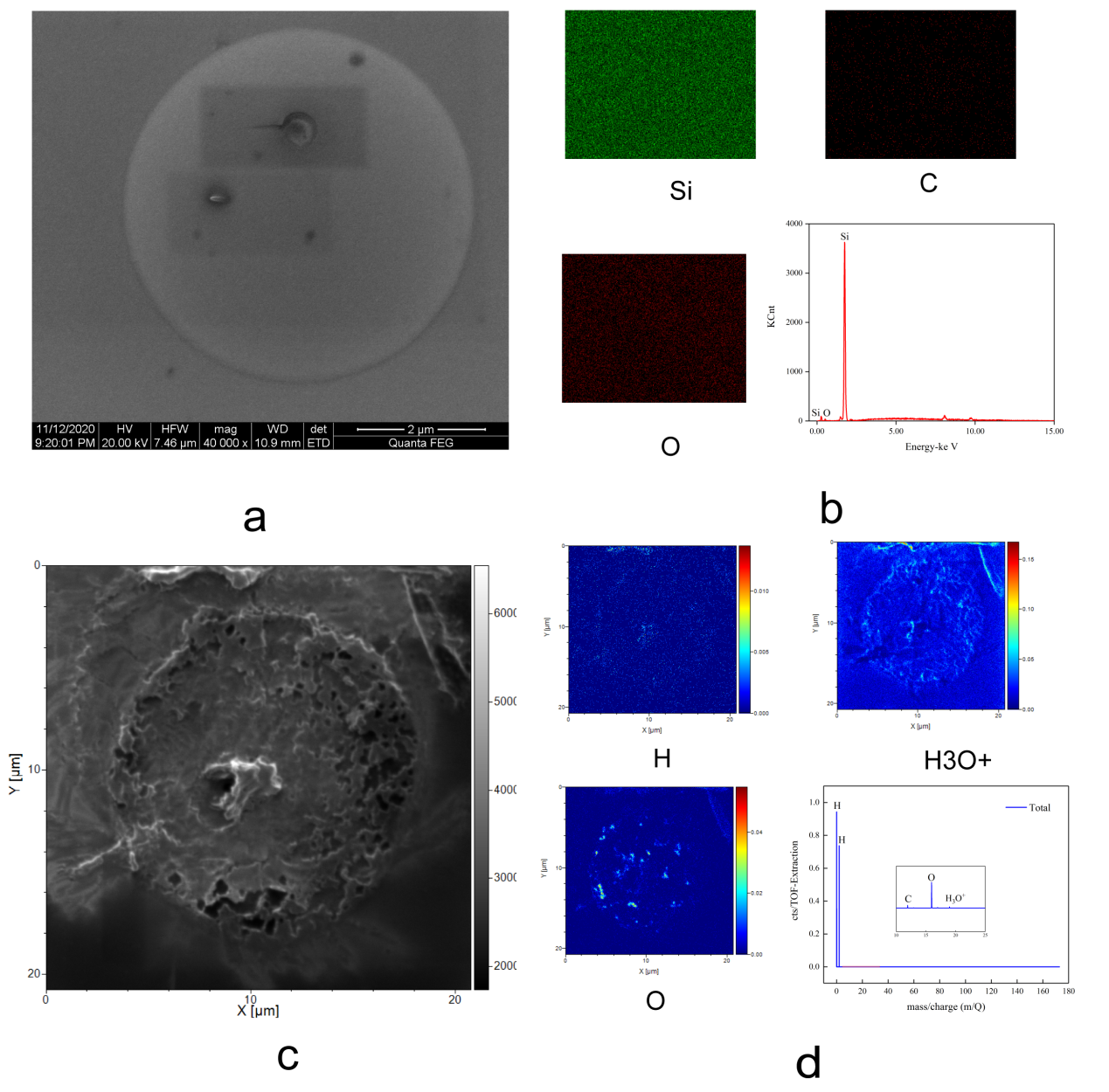


**Figure S8. Elemental and mass-spectrometric characterization of solid-like water (SSW) extruded from a SiO₂ microtubule (ID = 0.5 μm).**

1. SEM image of SSW extruded from a SiO₂ microtubule with an inner diameter of 0.5 μm. (b) EDS elemental maps (Si, C, and O) and the corresponding energy spectrum acquired from the same region as in (a). (c) SEM image of extruded SSW obtained by SEM–FIB–ToF-SIMS after storage under partial vacuum for three weeks. (d) ToF-SIMS ion maps of H, H₃O⁺, and O acquired from the same region as in (c), together with the corresponding mass spectrum. The data indicate that the extruded material is dominated by hydrogen- and oxygen-containing species, with only trace carbon contamination.

**Figure S9**


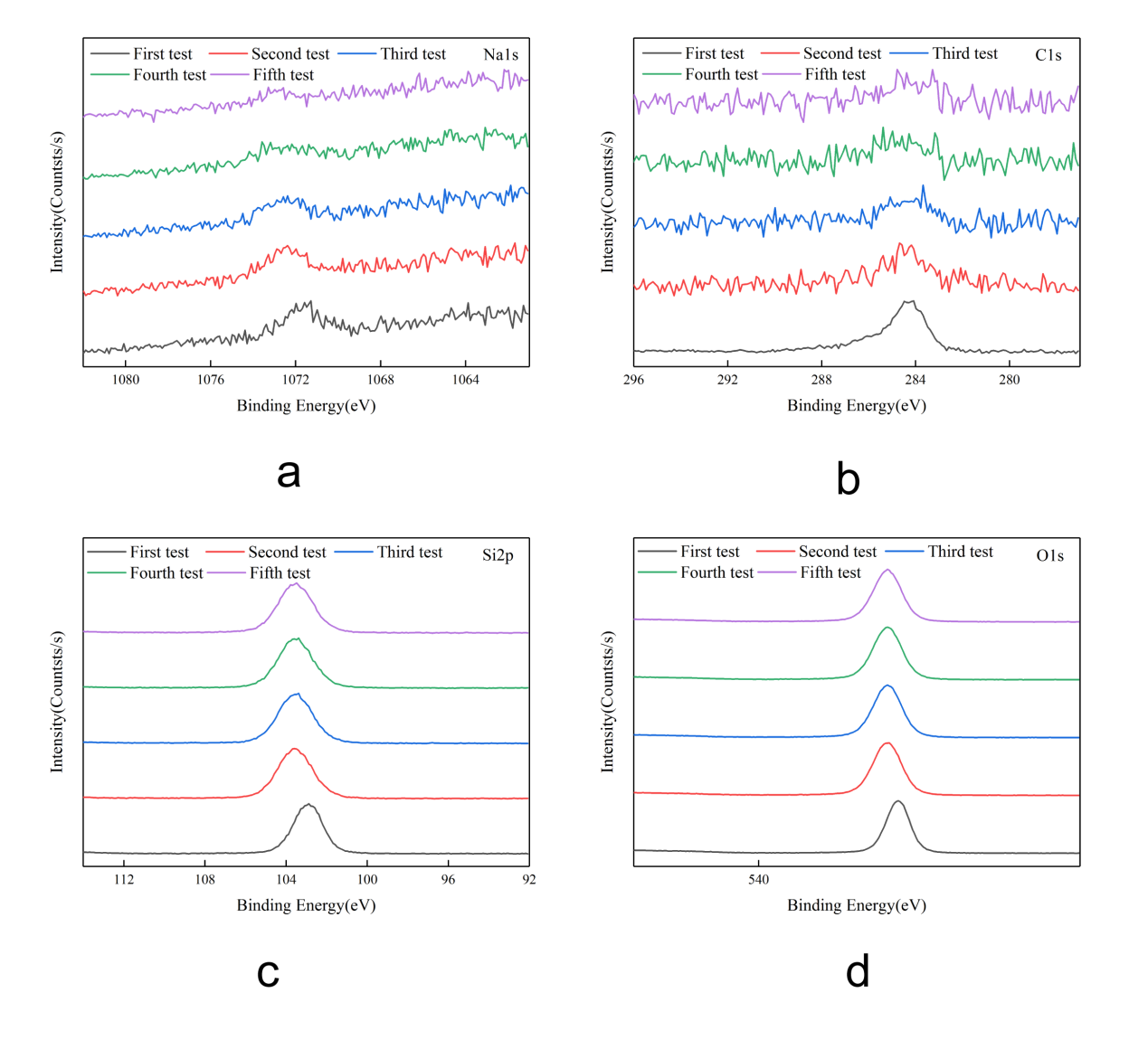


**Figure S9. X-ray photoelectron spectroscopy (XPS) depth profiling of the SiO₂ microtubule matrix (ID = 1 μm).**

XPS measurements were performed at the same location after immersing the microtubule in NaOH solution for 4 h. Each acquisition probes an estimated depth of ~2 nm. (a–d) XPS spectra of Na, C, Si, and O as a function of increasing analysis depth. Sodium is detectable only in the near-surface region and is no longer observed beyond ~6 nm. Carbon is primarily detected at the outermost surface and decreases rapidly with depth. In contrast, the Si and O signals remain essentially constant with increasing depth, indicating a chemically stable SiO₂ matrix beneath the surface layer.

**Figure S10**





**Figure S10. Changes in the total silanol content of SiO₂ microtubules with different inner diameters after surface treatment.**

Bar plots compare SiO₂ microtubules with inner diameters of 1, 5, and 100 μm. The y-axis represents the integrated ¹H NMR peak area of the SiOH signal, used as a measure of total silanol content. Orange and green bars denote the silanol content before and after surface modification, respectively. Surface modification was achieved by NaOH etching (decreasing SiOH density) and H₂O₂ treatment (increasing SiOH density).

**Figure S11**


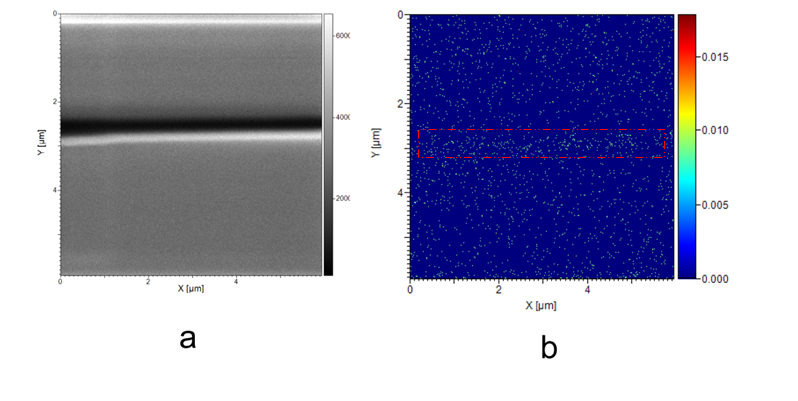


**Figure S11. Spatial distribution of hydrogen on the inner wall of a SiO₂ microtubule (ID = 1 μm).**

(a) SEM image of an empty microtubule longitudinally sectioned by SEM-FIB-ToF-SIMS. The dark band marks the microtubule lumen, while the surrounding lighter region corresponds to the silica wall. (b) Areal map of the H signal acquired from the same region as in (a). The color scale (right) represents the relative hydrogen intensity from low (blue) to high (red), and the dashed red outline indicates the location of the inner wall. A clear enrichment of hydrogen is observed near the inner surface compared with the bulk silica matrix, consistent with preferential localization of silanol groups at the microtubule inner wall.

**Figure S12**





**Figure S12. Depth-dependent silanol density in SiO₂ microtubules determined by ¹H NMR.**

Bar charts show the silanol density in microtubules with inner diameters of 1 μm and 5 μm, estimated from the integrated area of the SiOH resonance peaks in the ¹H NMR spectra (see Fig. S10). The y-axis shows the relative silanol density on a logarithmic scale. Orange bars correspond to the interfacial layer within 5 nm of the inner wall, whereas green bars represent regions deeper than 5 nm from the surface. A pronounced enrichment of silanol groups is observed in the near-surface layer, particularly for the 1 μm microtubule.

**Figure S13**


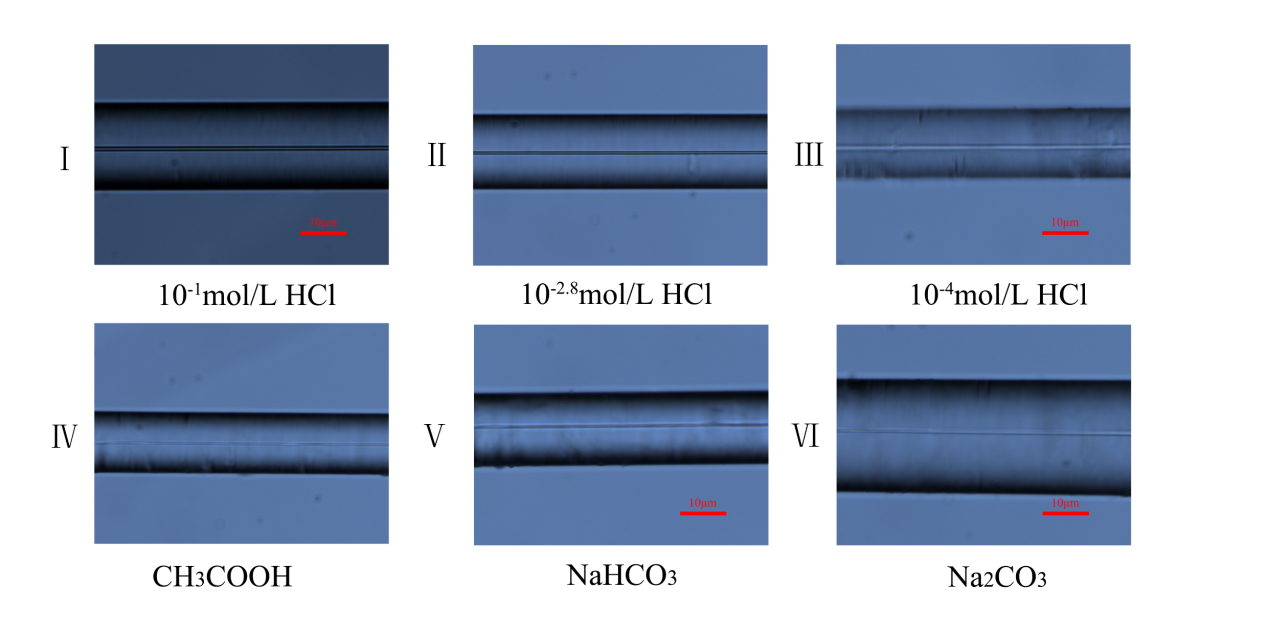


**Figure S13. Optical micrographs of solutions with different pH values confined in a SiO₂ microtubule (ID = 1 μm) after vacuum exposure.**

Optical micrographs were recorded after injecting aqueous solutions with different pH values into a SiO₂ microtubule (ID = 1 μm) and exposing the samples to vacuum for 10 min. Panels I–VI correspond to: (I) HCl (10⁻¹ mol L⁻¹), (II) HCl (10⁻².⁸ mol L⁻¹), (III) HCl (10⁻⁴ mol L⁻¹), (IV) acetic acid solution, (V) sodium bicarbonate solution, and (VI) sodium carbonate solution.

**Figure S14**


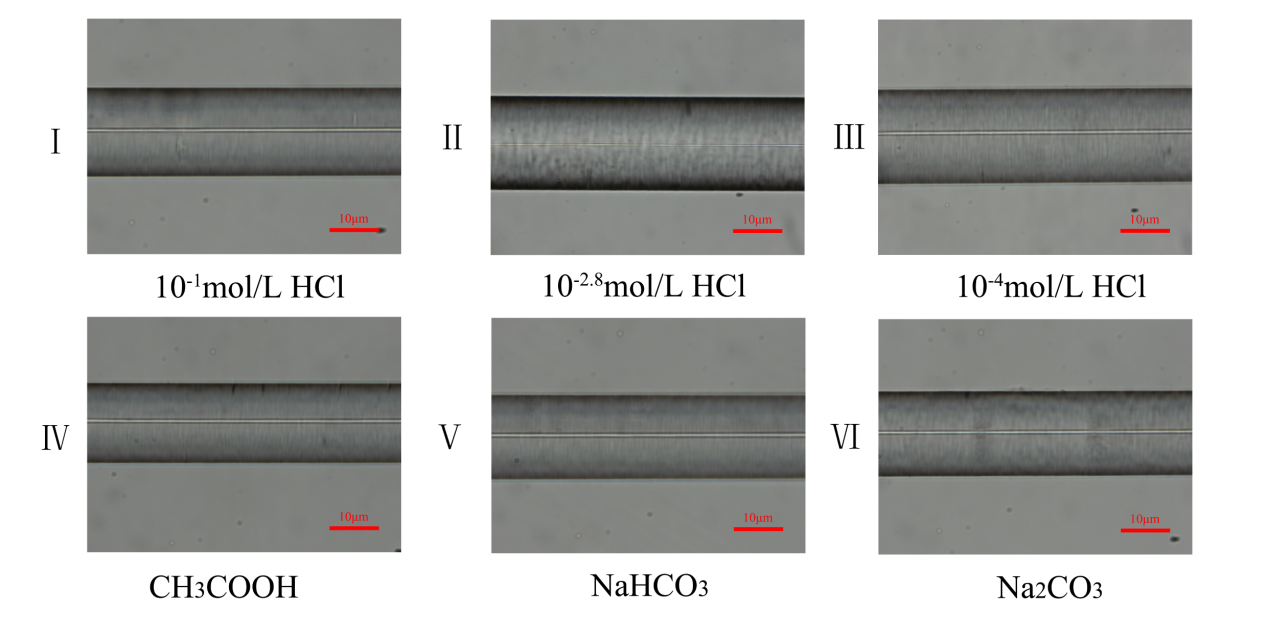


**Figure S14. Optical micrographs of NaCl solutions with different concentrations confined in a SiO₂ microtubule (ID = 1 μm) after vacuum exposure.**

Optical micrographs were acquired after injecting aqueous NaCl solutions with different concentrations into a SiO₂ microtubule (ID = 1 μm) and exposing the samples to vacuum for 10 min. Panels I–VI correspond to NaCl concentrations of 0.5, 1.5, 2.5, 3.5, 4.5, and 5.43 mol L⁻¹, respectively, corresponding to the conditions shown in Fig. 4e.
